# 

**Published:** 1789

**Authors:** 


### THE LONDON MEDICAL JOURNAL,

FOR THE YEAR 1789,

PART THE SECOND.

### CATALOGUE OF BOOKS.

1. AN Introduction to the Practice of Midwifery. By *Thomas Denman*, M. D. Licentiate in Midwifery of the College of Physicians. Volume the First. 8vo. *Johnson*, London, 1788.

2. Meteorological Account of the Weather at Madras from the 1st of June, 1787, to the 31st of May, 1788. 8vo. Madras, 1788.

3. A Treatiſe on Female, Nervous, Hyſterical, Hypochondriacal, Bilious, Convulſive Diſeaſes; Apoplexy and Palſy; with Thoughts on Madneſs, Suicide, &c. In which the principal Diſorders are explained from anatomical Facts, and the Treatment formed on ſeveral new Principles. By *William Rowley*, M. D. Member of the Univerſity of Oxford, the Royal College of Phyſicians, &c. 8vo. *Hookham*, London, 1788.

4. A Treatiſe on the Prevention of Diſeaſes incidental to Horſes from bad Management in regard to Stables, Food, Water, Air, and Exerciſe. To which are ſubjoined, Obſervations on ſome of the ſurgical and medical Branches of Farriery. By *John Clark*, Farrier to His Majeſty for Scotland. 8vo. *Smellie*, Edinburgh, 1788.

5. A Treatiſe on the Materia Medica. By *William Cullen*, M. D. Profeſſor of the Practice of Phyſic in the Univerſity of Edinburgh; Firſt Phyſician to His Majeſty for Scotland; Fellow of the Royal College of Phyſicians of Edinburgh; of the Royal Societies of London and of Edinburgh; of the Royal Society of Medicine of Paris; of the Royal College of Phyſicians of Madrid; of the American Philoſophical Society of Philadelphia; of the Medical Society of Copenhagen; of the Medical Society of Dublin; of the Royal Medical and of the Royal Phyſico-Medical Societies of Edinburgh. 2 Vols. 4to. *Elliot*, London, 1789.

6. A Letter addreſſed to Dr. Prieſtley, Meſſieurs Cavendiſh, Lavoiſier, and Kirwan, endeavouring to prove that their newly-adopted Opinions of inflammable and dephlogiſticated Airs forming Water, and the Acids being compounded of the different Kinds of Air, are fallacious. By *Robert Harrington*, M. D. 8vo. *Faulder*, London, 1789.

7. A ſhort Appendix to Dr. *D. Monro*'s Treatiſe on Medical and Pharmaceutical Chemiſtry, and the Materia Medica. To which is added, an Anſwer to the Remarks of the Critical Review for October, 1788, on the Firſt Volume of that Work. 8vo. *Cadell*, London, 1789.

8. Vindication of the Opinions and Facts contained in a Treatiſe on the Glandular Diſeaſe of Barbadoes. By *James Hendy*, M. D. Member of the Edinburgh Royal Medical Society; Phyſician during the late War to His Majeſty's Naval Hoſpital at Barbadoes; Phyſician General to the Militia, and one of the

Phyſicians

Phyficians to the General Difpenfary of the Ifland. 8vo. *Kearfley*, London, 1789.

9. A General Syftem of Chemiftry, theoretical and practical; digefted and arranged with a particular View to its Application to the Arts. Taken chiefly from the German of M. *Wiegleb*. By *C. R. Hopfon*, M. D. 4to. *Robinfons*, London, 1789.

10. Synopfis of the Natural Hiftory of Great Britain and Ireland; containing a fyftematic Arrangement and concife Defcription of all the Animals, Vegetables, and Foffils, which have hitherto been difcovered in thefe Kingdoms. By *John Berkenhout*, M. D.; being a fecond Edition of the Outlines, &c. corrected and confiderably enlarged. 8vo. 2 Vols. *Cadell*, London, 1789.

11. A Comparative View of the Phlogiftic and Antiphlogiftic Theories, with Inductions. To which is annexed, an Analyfis of the Human Calculus, with Obfervations on its Origin, and on the moft probable Means of preventing its ~~Prevention~~ formation. By *William Higgins*, of Pembroke College, Oxford. 8vo. *Murray*, London, 1789.

12. A few Obfervations concerning thofe Things which are probable, or in fome Meafure afcertained, aſcertained, relative to the Hiſtory and Cure of the Plague. By *William Henderſon*, M. D. Member of the Royal Medical Society of Edinburgh. 8vo. *Murray*, London, 1789.

13. An Inquiry into the Nature, Cauſes, and Termination of Nervous Fevers; together with Obſervations tending to illuſtrate the Method of reſtoring His Majeſty to Health, and of preventing Relapſes of his Diſeaſe. By *Robert Jones*, M. D. and Member of the Royal Society of Antiquaries at Edinburgh. 8vo. *Collins*, Saliſbury, 1789.

14. Vaſorum Lymphaticorum Corporis humani Hiſtoria et Ichnographia. Auctore *Paulo Maſcagni*, in Regio Senarum Lyceo publico Anatomes Profeſſore. Fol. max. Senis, 1787. c. Tab. æn. xxvii.

15. *Baſſiani Carminati*, Phil. et Med. Doct. in Ticinen. Gymnas. Hygien. Therap. Mat. Med. et Chirurg. ac Pharmac. R. Prof. Noſocom. Med. et var. Acad. Sodalis Opuſcula Therapeutica. Vol. I. 8vo. Pavia, 1788.

16. *Joſephus Gaertner*, M. D. Acad. Imp. Scient. Petrop. Memb. et Reg. Soc. Scient. Lond. Sodal. de Fructibus et Seminibus Plantarum. Accedunt Seminum Centuriæ quinque priores cum cum Tabulis æneis lxxix. 4to. Stutgardiæ, 1788.

17. Tractatus Botanico-Medicus de Achilleis; cui accedit Supplementum generis Tanaceti. Auctore *Carolo Ludovico Willdenow*, Med. Doct. Societ. Nat. Curiof. Halens. Sodal. 8vo. Halæ Magdeburgicæ, 1789.

18. Quod Cogitant Auctores de Hymene, et de Signis Virginitatis diverfis, et quod cogitari poteft. Auctore *J. L. M. Guillemeau*, Niortenfi, Dioecefis Pictavienfis. 8vo. Monfpelii, 1788.

19. *Maximiliani Stoll*, Medicinæ Clinicæ P. p. o. in Univerfitate Vindobonenfi, Prælectiones in diverfos Morbos chronicos, poft ejus obitum edidit et præfatus eft *Jof. Eyerel*. 8vo. Vindobonæ, 1788.

20. Obfervatio Medico-practica Febris puerperarum cum manifefta lactis in cavum Abdominis metaftafi adjuncta Epicrifi. Auctore *D. J. G. Zehner*. 4to. Manheim, 1787.

21. *Georg. Rud. Boehmeri*, Prolufio quâ Cyani fegetum nuper expertæ vires laudantur. 4to. Lipfiæ, 1787.

22. Thefaurus pathologico-therapeuticus; exhibens fcripta rariora et felectiora auctorum et indigenorum et exterorum, quibus natura ac medela medela morborum tam internorum quam externorum illuſtrantur atque explicantur; quem collegit et edidit D. *Jo. Chr. Traug. Schlegel*, Celſ. Comit. regn. de Schoenburg Waldenburg Conſil. et Archiater, &c. Volum. I. Pars I. 8vo. Lipſiæ, 1789.

23. Diſſertationes Medicæ in Univerſitate Vindobonenſi habitæ ad Morbos chronicos pertinentes et ex *Maximiliani Stollii*, Medicinæ Clinicæ P. p. o. prælectionibus potiſſimum conſcriptæ. Edidit et præfatus eſt *Joſephus Eyerel*. Vol. I. 8vo. Viennæ, 1788.

24. *I. G. Gunzii*, Phil. et Med. Doct. &c., de Cortice Salicis Cortici Peruviano ſubſtituendo, Commentatio. 8vo. Lipſiæ, 1787.

25. *Henrici Calliſen* Principia Syſtematis Chirurgiæ hodiernæ. Pars prior. 8vo. Havniæ, 1788.

26. Sylloge Selectiorum Opuſculorum * de Mirabili Sympathia, quæ partes inter diverſas

* Viz. 1. *J. P. Michell* de mirabili quæ caput inter et partes generationi dicatas intercedit, ſympathia; 2. *P. Jas*, de mirabili, quæ pectus inter et ventriculum intercedit, ſympathia; 3. *D. Vergens*, de ſympathia inter ventriculum et caput præcipue in ſtatu præternaturali; & 4. *J. Anemaet*, de mirabili quæ mammas inter et uterum intercedit, ſympathia.

corporis corporis humani intercedit, edita curâ *Jo. Chr. Traugott Schlegel*, Doct. Medic. atque Chir. et Medici apud Longosalissenses. 8vo. Lipsiæ, 1787.

27. Supplemento alla Memoria * per servire alla facile e perfetta estinzione del Vajuolo, e di tutti gli altri morbi contagiosi si acuti, che cronici, eccetuata la Lue Venerea, in tutta l'Europa, e nelle altre Nazioni, presso le quali non nascono endemici, del Sacerdote e Dottore in Filosofia e Medicina D. *Francesco Maria Scuderi* di Viagrandre presso Catania, oggi, per Real ordine, emanato dal suo graziosissimo Sovrano Ferdinando IV. nel di 21 Settembre 1787, Protomedico dell' Universita de' Regi generali Studj della suddetta chiarissima e fedelissima Citta di Catania, ed in tutto il Regno di Sicilia, e sue Isole adjacenti. A gui si aggiunge Apparatus Institutionum pathologico-practicarum, a magni Hippocratis doctrina majori ex parte sumptarum. 8vo. Napoli, 1788.

28. Delle Facoltà dell' Oppio nelle Malattie Veneree, nuove ricerche di *Giuseppe Pasta*. 8vo. Bergamo, 1788.

* See Vol. IX. page 217.

 29. Es-

29. Eſſais ou Recueil de Memoires ſur pluſieurs Points de Mineralogie, avec la deſcription des pieces depoſées chez le Roi, la figure, et l'analyſe chimique de celles qui ſont les plus intereſſantes, et la Topographie de Moſcow; après un Voyage fait au Nord par ordre du Gouvernement. Par M. *Macquart*, Docteur Regent de la Faculté de Medecine de Paris, Membre de la Société Royale de Medeçine, &c. 8vo. Paris, 1789.

30. Diſſertation ſur les Avantages des nouvelles Dents et Rateliers artificiels, incorruptibles et ſans odeur, inventés par M. *de Chemant*, Chirurgien et Dentiſte, et approuvés par la Faculté et par la Société Royale de Medecine de Paris; ſuivie d'une refutation des aſſertions avancées par M. *Dubois Foucon*, Dentiſte du Roi en ſurvivance, dans ſa lettre aux Auteurs du Journal de Paris, le 18 Mai, 1788. 8vo. Paris, 1789.

31. Obſervations generales ſur les Eaux de Cheltenham, par *J. Smith*, M. D. Profeſſeur Savilien de Geometrie dans l'Univerſité d'Oxford; precedées de diverſes analyſes, citations de pluſieurs medecins Anglois, ſur l'uſage de ces eaux: traduites de l'Anglois par M. *F. Le Breton*, Inſpecteur General des Remiſes des Capitaineries ries Royales, Membre de l'Academie Royale des Sciences d'Upſal, &c. &c. 8vo. Paris, 1789.

32. Cours de Botanique pour ſervir a l'Education des Enfans de S. A. S. Monſeigneur le Duc d'Orleans ; ou l'on a raſſemblé les Plantes indigenes et exotiques employées dans les Arts et dans la Medecine. Par M. *Alyon*, Lecteur de S. A. Sereniſſime Monſeigneur le Duc de Chartres. Folio. Paris, 1789.

33. Traité Elementaire de Chimie, preſenté dans un ordre nouveau et d'après les Decouvertes modernes; avec Figures. Par M. *Lavoiſier*, de l'Academie des Sciences, de la Société Royale de Medecine, des Sociétés d'Agriculture de Paris et d'Orleans, de la Société Royale de Londres, de l'Inſtitut de Bologne, de la Société Helvetique de Baſle, de celles de Philadelphie, Harlem, Mancheſter, Padoue, &c. 2 Tomes. 8vo. Paris, 1789.

34. Eloge Hiſtorique de *Pierre Richer de Belleval*, Inſtituteur du Jardin Royal de Botanique de Montpellier, ſous Henri IV. Memoire qui a remporté le Prix de la Société Royale des Sciences en 1788. Par M. *Dorthes*, Docteur-Medecin de la Faculté de Montpellier, Membre de la Société Royale des Sciences, Correſpon-

 dant dant de la Société Royale d'Agriculture de Paris, &c. 4to. Montpellier, 1788.

35. Aſſemblée publique de la Société Royale des Sciences, tenue dans la Grande Salle de l'Hotel de Ville de Montpellier, en preſence des Etats de la Province de Languedoc, le 12 Janvier, 1788. 4to. Montpellier, 1788.

36. Hiſtoire de la Société Royale de Medecine. Années 1784 & 1785. Avec les Memoires de Medecine et de Phyſique Medicale pour les memes années, tirés des Regiſtres de cette Société. 4to. Paris, 1788.

37. Chemiſche Unterſuchung einiger der bekanntern und beſuchtern Geſundbrunnen und Baeder der Schweitz, inſbeſondre des Cantons Bern; nebſt einer Beſchreibung der neueſten Unterſuchungs Methoden; durch eigene Erfahrungen vermehrt und beſtattigt, &c. *i. e.* A chemical Examination of ſome of the moſt known and frequented Mineral Waters and Baths of Switzerland, particularly of the Canton of Bern; with a Deſcription of the neweſt Methods of proſecuting ſuch Inquiries: Improved and confirmed by Experiments made by the Author, *C. F. Morell,* Apothecary at Bern. 8vo. Bern, 1788.

38. Hand-

38. Handbuch der Apothekerkunſt nach der neueſten Entdeckungen, &c. *i. e.* A Manual of Pharmacy according to the lateſt Diſcoveries. By *J. P. Steyrers*, M. D. Vol. I. 8vo. Salzburg, 1787.

39. Von der Wuerkung und dem Einfluſſe der Einbildungſkraft der Mutter auf die frucht; aus gruenden und haeufigen Erfahrungen bewieſen; *i. e.* Of the Effect and Influence of the Mother's Imagination on the Fœtus; confirmed by numerous and well-founded Experiments. By *K. C. Krauſe*, M. D. Profeſſor of Phyſic at Leipſic. 8vo. Leipſic, 1787.

40. Abhandlungen und Beobachtungen aus der practiſchen und gerichtlichen Arzneywiſſenchaft; *i. e.* Eſſays and Obſervations relative to the Practice of Phyſic and to Medical Juriſprudence. By *J. F. Keck*, M. D. Phyſician to the Prince of Anhalt-Zerbſt at Coſwig. 8vo. Berlin, 1787.

41. Vermiſchte Mediciniſche Schriften; *i. e.* Miſcellaneous Medical Eſſays, by *K. A. Zwierlein*, M. D. Inſpector of the Mineral Waters and Phyſician at Brueckenau. 8vo. Heidelberg, 1788.

42. Mediciniſche Fragmente aus der verlaſſenſchaft des D. *Thom. Knigge* in Regenſburg nebst dessen lebenslauf, &c. *i.e.* Medical Fragments by the late Dr. *Thomas Knigge* of Ratisbon; with an Account of his Life. By *Joh. Jac. Kohlhaas*, M. D. 8vo. Ratisbon, 1788.

43. Geschichte einer Zwillings Kaysergeburt; *i. e.* An Account of a Delivery of Twins by the Cæsarean Operation. By *J. C. Sommer*, M. D. 8vo. Leipsic, 1788.

44. Trattato delle Malattie esterne del Cavallo, di *Francesco Toggia*, Regio Veterinario. Tom. II. 8vo. Vercelli, 1788.

45. Dissertatio Chemica Medicamentorum Antimonialium conspectum sistens; Auctore *Joanne Moeller*, M. D. 8vo. Hafniæ, 1787.

46. Descriptio nervi Ischiadici; Auctore *Joanne Henrico Joerdens*, M. D. Folio. Erlangen, 1788. c. Tab. æneis v.

47. *Ludovici Francisci Maincourt*, Doctoris Medici Andegavensis, Dissertatio Medico-physica de Sanguineis lymphaticisque, malé polypis dictis, concretionibus, in corde et in vasis, per vitam existentibus. 8vo. Lutetiæ Parisiorum, 1789.

48. Exercitationum physicarum de causis physicis miræ illius tùm in homine, tùm inter homines, tùm denique inter cætera Naturæ corpora pora ſympathiæ, Diſſertationes duæ, Auctore *J. H. Rahn.* 4to. Zurich, 1786-8.

49. Vermiſchte Botaniſche Abhandlungen; *i. e.* Miſcellaneous Botanical Eſſays by *J. G. Gleditch*; collected and publiſhed, with a Preface, by *D. R. A. Gerhard.* Vol. I. 8vo. Berlin, 1789.

50, Grundritz der Allgemeinen Chemie zum gebrauch bey Vorleſungen; *i. e.* Principles of General Chemiſtry, intended as a Text Book for Lectures, by *J. F. Gmelin.* Part I. 8vo. Goettingen, 1789.

51. Giornale Scientifico, Letterario e delle Arti, di una Societa Filoſofica di Torino; raccolto e poſto in ordine da *Gioanni Antonio Giobert* e Dottor *Carlo Giulio*, Membri di varie Accademie. Tom. I. 8vo. Turin, 1789.

52. De Poena Funis, ſeu de Funis Ictuum Atrocitate et Periculis, de que advertendis a Medico et Chirurgo in eorum examine qui tali poenæ ſunt ſubjiciendi, Animadverſiones *Nicolai Agnelli*, Ferrarienſis. 8vo. Ferrara, 1788.

53. Pharmacopœa in uſum Noſocomii Fridericiani Hafnienſis edita a *Fr. L. Bang.* 12mo. Hafniæ, 1788.

54. Conſeils pour les Femmes de quarante cinq a cinquante ans, ou Conduite a tenir lors de de la Ceſſation des Regles; par le celebre praticien de Londres, le Docteur *Fothergill*: Extrait des Obſervations et Recherches de la Société Medicale de Londres. 8vo. Paris, 1788.

55. Memoire qui a Remporté le Prix, au Jugement de la Faculté de Medecine de Paris, le 29 Decembre, 1785, ſur la queſtion propoſée en ces termes: — "Decrire l'ictere des "nouveau-nés, et diſtinguer les circonſtances "où cet ictere exige les ſecours de l'art, et "celles où il faut tout attendre de la nature?" Par M. *Baumes*, Docteur en Medecine de la Faculté de Montpellier, agrégé au College des Medecins de Niſmes; Medecin de l'Hoſpice de Charité de la meme Ville; Aſſocié Regnicole de la Société Royale de Medecine de Paris, &c. 8vo. Niſmes, 1788.

56. Epitre à Meſſieurs les Savans et Amateurs en Chimie, pour ſervir de reponſe à un article des Elemens d'Hiſtoire Naturelle et de Chimie de M. *Fourcroy*; ſuivie de pluſieurs Memoires ſur les Operations nouvelles et curieuſes en Chimie; par M. le Baron de *Bormes*. 8vo. Paris, 1787.

